# Administering Virtual Reality Therapy to Manage Behavioral and Psychological Symptoms in Patients With Dementia Admitted to an Acute Care Hospital: Results of a Pilot Study

**DOI:** 10.2196/22406

**Published:** 2021-02-03

**Authors:** Lora Appel, Erika Kisonas, Eva Appel, Jennifer Klein, Deanna Bartlett, Jarred Rosenberg, Christopher NC Smith

**Affiliations:** 1 Faculty of Health School of Health Policy and Management York University Toronto, ON Canada; 2 OpenLab University Health Network Toronto, ON Canada; 3 Michael Garron Hospital Toronto, ON Canada

**Keywords:** virtual reality, wearable electronic devices, sensory art therapies, hospitalization, hospitals, community, hospitals, general, aged, humans, dementia, behavioral symptoms, nature, mobile phone

## Abstract

**Background:**

As virtual reality (VR) technologies become increasingly accessible and affordable, clinicians are eager to try VR therapy as a novel means to manage behavioral and psychological symptoms of dementia, which are exacerbated during acute care hospitalization, with the goal of reducing the use of antipsychotics, sedatives, and physical restraints associated with negative adverse effects, increased length of stay, and caregiver burden. To date, no evaluations of immersive VR therapy have been reported for patients with dementia in acute care hospitals.

**Objective:**

This study aimed to determine the feasibility (acceptance, comfort, and safety) of using immersive VR therapy for people living with dementia (mild, moderate, and advanced) during acute care hospitalization and explore its potential to manage behavioral and psychological symptoms of dementia.

**Methods:**

A prospective, longitudinal pilot study was conducted at a community teaching hospital in Toronto. The study was nonrandomized and unblinded. A total of 10 patients aged >65 years (mean 86.5, SD 5.7) diagnosed with dementia participated in one or more research coordinator–facilitated sessions of viewing immersive 360° VR footage of nature scenes displayed on a Samsung Gear VR head-mounted display. This mixed-methods study included review of patient charts, standardized observations during the intervention, and pre- and postintervention semistructured interviews about the VR experience.

**Results:**

All recruited participants (N=10) completed the study. Of the 10 participants, 7 (70%) displayed enjoyment or relaxation during the VR session, which averaged 6 minutes per view, and 1 (10%) experienced dizziness. No interference between the VR equipment and hearing aids or medical devices was reported.

**Conclusions:**

It is feasible to expose older people with dementia of various degrees admitted to an acute care hospital to immersive VR therapy. VR therapy was found to be acceptable to and comfortable by most participants. This pilot study provides the basis for conducting the first randomized controlled trial to evaluate the impact of VR therapy on managing behavioral and psychological symptoms of dementia in acute care hospitals.

## Introduction

### Background

The term behavioral and psychological symptoms of dementia (BPSD) encompass a range of manifestations, including agitation, aggression, delusions, hallucinations, depression and apathy, sleep troubles, and wandering. These symptoms are complex, costly to treat, and lead to poor health outcomes; they are distressing both for people living with dementia and those who care for them [1]. For patients with dementia admitted to an acute care inpatient unit, the prevalence of associated BPSD is up to 75%, with aggression and activity disturbance being the most common [2,3]. Family caregivers have given rich reports about how BPSD may worsen during the acute care hospital stay and how hospital staff struggle to manage these symptoms adequately [2].

Current interventions to manage BPSD include pharmacological interventions, such as neuroleptic or sedating medications, and the application of physical barriers such as bed alarms, locks, Buxton chairs, and tethers. Many harmful consequences have been associated with pharmacological interventions, including cardiovascular events, falls, hastening of cognitive decline, and death [3]. Physical restraints are not any more benign, increasing the risks of pressure sores and infection and worsening anxiety, distress, and acts of physical violence; their use poses ethical and acceptability issues. New approaches are clearly needed, and many nonpharmacologic strategies have been attempted with varying levels of success. Following in the footsteps of music therapy and arts-based therapy [4-8], researchers have envisioned the application of virtual reality (VR) technology for dementia care [9-11].

VR is the term used to describe a 3D, computer-generated environment, which can be viewed and/or interacted with using special equipment such as head-mounted displays (HMDs) and haptic gloves to synchronously stimulate our senses to create the illusion of reality. VR provides a unique opportunity to expose individuals who are otherwise confined indoors (eg, in hospitals) to a variety of simulated natural and social environments that can be both calming and engaging (eg, peaceful beach, sunny autumn forest, and live music at a restaurant). Experiencing these environments may reduce symptoms such as depression and anxiety [12,13]. VR has been used in health care since the 1990s, developing into a tool that can address a variety of mental health concerns. The customizability of the illusion of reality created by VR environments has enabled clinicians to treat social anxiety disorders, specific phobias, eating disorders, substance misuse disorders, and depression using VR [14,15]. It has also been used in medical training, poststroke rehabilitation, and pain management [14,15]. Researchers and clinicians are eager to explore the potential of VR therapy to manage BPSD in the hope that this may prove to be a less expensive, noninvasive, and ethically acceptable means of engaging and distracting individuals with dementia, without the negative adverse effects associated with current approaches (eg, medication and physical restraints) [16,17].

Recent articles have reported a growing number of success stories of administering VR therapy to people living with dementia to alleviate stress, depression, and anxiety [18-21]. However, VR has yet to be rigorously evaluated as a type of therapy in various settings, including community care in private residences, rehabilitation centers, long-term care institutions, and acute care hospitals. We conducted a multisite feasibility study with 66 participants with varying severities and types of dementia or cognitive impairment recruited from a rehabilitation center, long-term care institution, or outpatient clinic day program [22]. On the basis of positive results of this study (ie, 85% of participants found the HMD easy to wear, 71% of participants would recommend VR therapy to a friend, and 76% of participants wanted to try VR therapy again) and a strong interest in nonpharmacologic solutions to help deprescribe antipsychotics for the management of BPSD from hospital clinicians, we designed a randomized controlled trial (RCT) to evaluate the impact of VR therapy on patients with dementia admitted to an acute care hospital. The design of the RCT was informed by this pilot study, which was reported in 2 papers. The first described protocol changes to processes, methods, workflow for the subsequent RCT, and learnings for introduction of a nonpharmacological intervention to an acute care hospital setting [23]. Second, this study reports on the feasibility (ie, acceptance, comfort, and safety) of administering immersive VR therapy (IVR) to patients with dementia in an acute care hospital.

### Aims

The primary aim of the pilot study was to determine the feasibility of administering IVR therapy to inpatients at an acute care hospital in various stages of dementia, particularly those in moderate and advanced stages. This included assessing the tolerability or acceptability, comfort, and safety of the VR equipment and 360° film experiences. As an additional exploratory objective, the study looked at the potential of VR therapy to reduce the frequency and/or intensity of participants’ BPSD during hospitalization.

## Methods

### Design

This study was a prospective, longitudinal pilot study. The study was nonrandomized and unblinded, and all participants received the study intervention.

### Setting

The pilot study was conducted at a community teaching hospital affiliated with the University of Toronto, Toronto, Canada. Participants were recruited from the General Internal Medicine Department between July and October 2018. Ethics approval for the pilot study was obtained from the hospital research ethics board (reference number 748-1806-Mis-321).

### Participants and Informed Consent

A total of 516 prospective participants were screened for possible inclusion in this study between July 31 and October 31, 2018. Of the 516 participants, 67 were eligible, and a total of 10 participants with dementia were recruited. Multimedia Appendix 1 shows the recruitment process, which is further discussed in detail in a second manuscript [23]. Where applicable, the substitute decision maker (SDM) of prospective participants was contacted over the phone and introduced to the study. The SDM was also often the patient’s primary caregiver. The research coordinator (RC) then employed a shared decision-making approach, whereby a signed informed consent (Multimedia Appendix 2) for the study was obtained from the SDM in person, and each study session was assented to by the patient. Although SDMs were permitted at study sessions, they were not formal participants in the study and therefore did not need to sign consent documents for themselves.

### Inclusion or Exclusion Criteria

Patients were included if they were (1) aged >65 years, (2) diagnosed with dementia, and (3) admitted as inpatients at the study-site hospital. Patients were excluded if they (1) had open facial wounds, (2) had cervical conditions that would make use of a VR headset unsafe, and (3) had no contactable SDM (if applicable).

### VR Therapy Intervention

**Figure 1:**
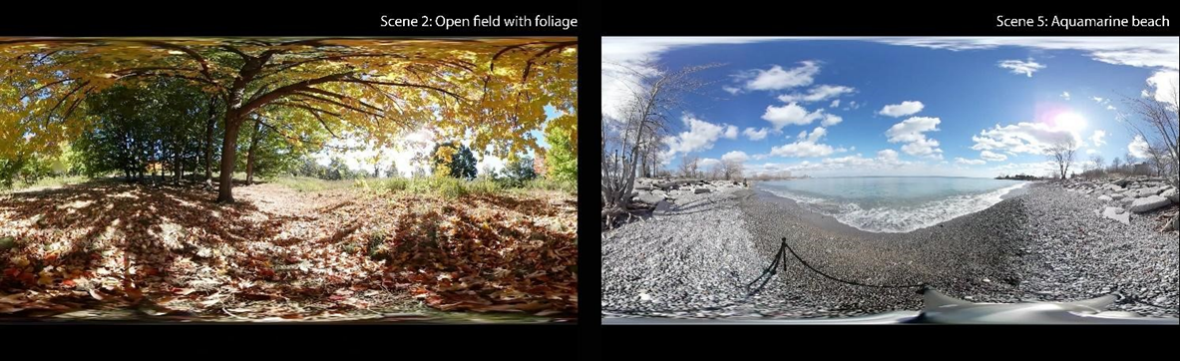
2D screen capture of 2 of the 5 virtual reality scenes (scene 2: open field with foliage and scene 5: Aquamarine beach).

**Figure 2:**
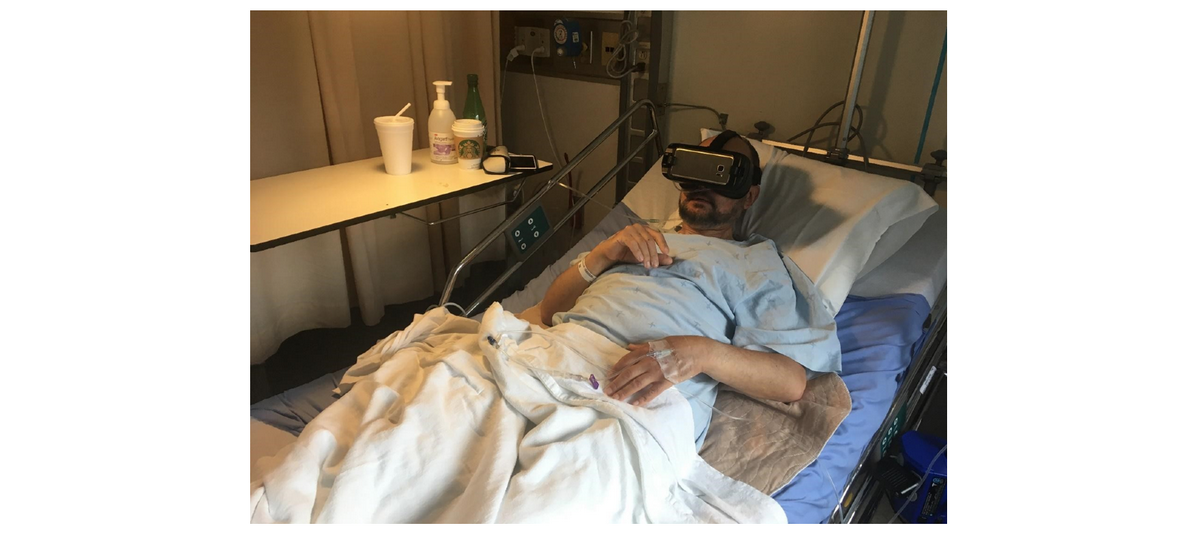
Participant tries the virtual reality experience. Written informed consent was obtained from the individual for the publication of this image.

The VR therapy intervention consisted of participants (individuals with dementia) viewing a sequence of 5 short 360° video clips (1-3 minutes each) depicting various natural scenes (rocky lakeshore, sunny forest, dense forest, floating icebergs, and sunny beaches) for a maximum of 20 minutes. Figure 1 shows a screenshot from 2 scenes. Participants could loop through the VR film sequence for up to 20 minutes. A nurse, SDM or caregiver, or RC helped them sit up in bed, and the RC assisted them to put on and remove the HMD. Figure 2 shows a patient trying the VR experience in a hospital bed.

Participants used a Samsung Gear VR HMD and Sennheiser HD 221 headphones. The HMDs were equipped with individual removable foam inserts to meet the hospital’s infection prevention and control hygiene requirements.

### Data Collection

#### BPSD at Baseline and During Hospitalization

BPSD at baseline was reported using the Neuropsychiatric Inventory (NPI; 12-item), a validated scale that measures the presence or level of BPSD and reflects changes in patient behavior since the onset of dementia [24]. The NPI was administered to a primary caregiver (which was also the SDM) who rated the patient’s behavior from the past 4-6 weeks. In-hospital BPSD was measured by counting the total number of instances of BPSD (by category) from the nursing notes during hospitalization.

On the basis of recommendations by a geriatrician, instances of BPSD were grouped into the following categories: agitation, refusing or declining medical care, violence, wandering, vocalizations, insomnia symptoms, mood symptoms, disorganized thoughts and content (paranoia), perceptual disturbances, additional falls precautions applied, security personnel called, sitters or personal support workers at the bedside for patient monitoring purposes, physical restraints used, and chemical restraints used.

#### Baseline Health and Hospital Metrics

Demographic and health history information was gathered through survey questions from the participant, occasionally with support from the caregiver or SDM, during the first study session. This included the level of education, marital status, sensory or mobility impairments, and the use of assistive devices (eg, wheelchair, walker, cane, glasses, and hearing aids). Participants were also asked about previous experiences with VR technology. Other personal health information, including age, sex, cognitive diagnoses (as described in physician notes), markers related to participant hospital care (length of stay [LoS], discharge disposition, and all-cause mortality), dose and frequency of sedatives (daily and as-needed) administered during the hospital stay, Confusion Assessment Method scores, physical restraint use, number of pressure ulcers, and number of falls, were obtained through review of participants’ electronic medical records (EMRs). Of note, because of the lack of standardization among the tools and infrequent recording of scores for instruments used to assess cognitive impairment (eg, Montreal Cognitive Assessment and Mini-Mental State Examination), in the patients’ hospital EMR, dementia severity and cognitive status were based on the terminology used by physicians in their notes (ie, mild, moderate, and advanced) [23].

#### VR Acceptability, Comfort, and Safety

As an overall measure of VR acceptability (tolerability), we reported the number of sessions in which the participants were able to wear the HMD and recorded how long the participants kept the HMD on to view the films. A modified version of the State-Trait Anxiety Inventory (STAI Y) [25] was used to collect information about the participants’ current state of anxiety pre- and postintervention. Post-VR therapy, open-ended questions were asked to capture feedback about any discomfort experienced; whether the HMD was too heavy, if it applied too much pressure on their head, face, or nose as well as sound quality and image focus. Safety was operationalized by the presence of adverse events or adverse effects.

A modified version of the Music in Dementia Assessment Scales (MiDAS) [26], developed and validated to evaluate music therapy for people with dementia, was completed by the RC to assess whether there were observable changes in the participant’s mood or behavior and engagement (eg, interest, response, and enjoyment) while exposed to VR therapy. The RC recorded (through written notes that were later transcribed) any vocalizations, changes in facial expressions, breathing patterns, gestures, body movements, level of activity, and impressions of participant relaxation or enjoyment interpreted through observations of reactions and/or elicitation of spontaneous conversations such as recounting stories or pleasant life memories. Caregiver or SDM feedback regarding participant response to the VR intervention was also recorded. This included caregiver or SDM insights as to why participants reacted in certain ways to certain VR films. Finally, participants were asked about preferences for future VR film content and if they would be interested in additional VR therapy sessions.

## Results

### Participants

A total of 10 patients (8 female) with a mean age of 86.5 (SD 5.7) years participated in the study. Dementia severity ranged from mild (2/10, 20%), moderate (1/10, 10%), and advanced (4/10, 40%), with some unspecified (3/10, 30%). Half (5/10, 50%) the participants lived at home—3 (30%) lived alone, 1 (10%) lived with family members, and 1 (10%) had an other arrangement. The other half (5/10, 50%) of the participants lived in senior housing—4 (40%) lived in long-term care or assisted living and 1 (10%) lived in a retirement home or independent living. The majority of participants (8/10, 80%) were not in a relationship—3 (3/10, 30%) were widowed, 1 (1/10, 10%) was single, 1 (1/10, 10%) was separated, and 1 (1/10, 10%) had an other arrangement. Of the 10 participants, 2 (20%) were married.

Of the 10 participants, 6 (60%) were diagnosed with delirium during their hospital stay and 2 (20%) were diagnosed with comorbid cognitive conditions—1 (10%) had depression and 1 (10%) had depression, anxiety, executive dysfunction, and sleep issues. The majority of participants (8/10, 80%) were free of major visual or auditory impairments; 8 (8/10, 80%) wore glasses and 2 (2/10, 20%) used hearing aids. All participants used some form of mobility aid. The vast majority (9/10, 90%) of participants had normal head mobility, and most (8/10, 80%) had limited body mobility. Multimedia Appendix 3 provides more details.

### BPSD at Baseline and During Hospitalization

Presentation of BPSD during hospitalization varied greatly: the majority of participants displayed agitation (8/10, 80%), refusal of medical care (6/10, 60%), wandering (6/10, 60%), vocalizations (7/10, 70%) and symptoms of insomnia (8/10, 80%) and required additional fall precautions applied by staff (7/10, 70%). Of the 10 participants, 2 (20%) displayed violent behavior and 3 (30%) required a sitter or patient care assistant and personal support worker at the bedside for patient monitoring purposes. Some participants also had chemical restraints (4/10, 40%) or physical restraints (3/10, 30%) administered during their hospital stay. Table 1 provides the baseline and in-hospital presentation of BPSD by participant.

**Table 1:** Behavioral and psychological symptoms of dementia at baseline and in-hospital (N=10).

Characteristics		Value
Total Neuropsychiatric Inventory score (12-item), mean (SD)		13 (8.87)
**Behavioral and psychological symptoms of dementia behavior displayed, n (%)**		
	Agitation	8 (80)
	Symptoms of insomnia	8 (80)
	Additional fall precautions applied by staff	7 (70)
	Vocalizations	7 (70)
	Wandering	6 (60)
	Refusal of medical care	6 (60)
	Mood symptoms (depression or anxiety)	3 (30)
	Required constant monitoring by sitter or personal care aide or personal support worker	3 (30)
	Violent behavior	2 (20)
	Perceptual disturbances	1 (10)
	Paranoia	0 (0)
Chemical restraints		4
Physical restraints		3

### Baseline Health and Hospital Metrics

The average hospital LoS for study participants was 11.1 (SD 7.2) days, which is 36% above the average LoS of 7 days for senior patients without dementia admitted to this hospital. Daily medications related to cognition, mental health, and sleep disorders were recorded; of the 10 participants, 4 (40%) were prescribed at least one cognition-enhancing medication (eg, galantamine, memantine), 4 (40%) were prescribed at least one antidepressant medication (eg, selective serotonin reuptake inhibitors, serotonin, and norepinephrine reuptake inhibitors), and 1 (10%) was prescribed at least one antipsychotic medication (eg, risperidone and quetiapine). Of 10 participants, 3 (30%) experienced falls during their hospital stay, of whom 1 (10%) had multiple falls without injury. Table 2 provides details on the factors related to hospital stay.

**Table 2:** Factors related to hospital stay (N=10).

Characteristics		Value
Length of stay (days), mean (SD)		11.09 (7.16)
**Discharge disposition, n (%)**		
	Home	4 (40)
	Transferred to another institution (Complex Continuing Care)	2 (20)
	Transferred to another institution (rehabilitation center)	1 (10)
	Transferred to another institution (unknown)	1 (10)
	Expired	1 (10)
**Number of daily medications prescribed, n (%)**		
	3-4	2 (20)
	1-2	5 (50)
	0	3 (30)
Number of participants who fell with injury, n (%)		1 (10)
Number of participants who fell without injury, n (%)		2 (20)
Number of pressure ulcers, n (%)		0 (0)
Number of participants readmitted within 30 days of discharge, n (%)		1 (10)

### VR Therapy Intervention

#### VR Acceptability, Comfort, and Safety

A total of 18 VR sessions were conducted with 10 participants. None of the participants kept the HMD on for the entire 20-min maximum allotted time. Table 3 details the reasons why the VR HMD was removed prematurely. Participants watched VR videos for an average of 6.2 (SD 5.5) minutes. After completing the first study session, most participants (7/10, 70%) opted for additional sessions during their hospital stay.

Participants who had body mobility limitations (8/10, 80%) experienced the VR sessions in their hospital bed, seated in Fowler’s position (an almost upright position). Participants without body mobility limitations (2/10, 20%) viewed VR sitting independently on the side of their bed (1/10, 10%) or in a nonswivel chair (1/10, 10%).

**Table 3:** Reasons for premature removal of virtual reality head-mounted display (N=18).

Characteristic		Value
**Sessions stopped before maximum allotted time, n (%)**		18 (100)
	Stopped as per participants choice without distress	12 (67)
	Stopped due to low interest	3 (17)
	Stopped due to head-mounted display slipping off	1 (6)
	Stopped due to participant falling asleep	1 (6)
	Stopped due to negative side effects	1 (6)

The majority (7/10, 70%) of participants reported that they found the headset comfortable. Of the 10 participants, 1 (10%) found the VR headset too heavy; they also mentioned that they would like to own VR at home if a lighter model were available. Moreover, 2 participants (20%) were unable to provide feedback regarding comfort. No participants reported feeling pressure on their nose from the HMD.

Of the 10 participants, 1 (10%) experienced negative side effects of self-limiting dizziness with mild nausea but no vomiting from the VR session. There were no reports of interference between the VR equipment and any medical devices (eg, hearing aids).

#### VR Impact on Enjoyment and Relaxation

We found that 6 of the 10 (60%) study participants had difficulty answering the pre- and postsurvey questions about their mood before and after VR therapy, and the RC often relied on caregiver input and participant body language to make educated estimations of participants’ moods that they recorded as being communicated by the participants themselves. Multimedia Appendix 4 provides the results of the mood questions for each of the 18 sessions. To consolidate the table, where the participant was unable to respond (response recorded as N/A), we provided the response noted by the caregiver or researcher. Precedence was always given to the participant's response*.* Multimedia Appendix 5 summarizes the instances across the 18 VR sessions in which patients were able to respond to the mood questions; patients were able to respond 53% of the time in the prequestions and 58% in the postquestions.

In the majority of sessions (14/18, 78%), participants made some substantial conversation or vocalizations. Although multiple participants simply described what they were seeing, one participant expressed interest and desire to engage with their (virtual) surroundings:

“look at the waves there!” “I’d love to be there and paddle” “all I need is a swimsuit” “I have to get in, it’s too cold” “the forest! The tall trees… and lots of weeds… I wonder if there’s any deer in there.”’

P8-1

In more than half of the VR therapy sessions (10/18, 56%), the RC perceived some substantial expression of enjoyment by the participant during the VR experience. Observed behaviors perceived as participant enjoyment included actively looking around and movements that suggested they were interacting with their environment, such as reaching out with hands or legs, pointing, waving, and wiggling toes. Participants also displayed enjoyment through laughter and verbal feedback such as: “I liked the pictures” and “well God gave us a beautiful world.” Of the 10 participants, 1 (10%) expressed substantial enjoyment with frequent laughing every 10 seconds, pointing at scenery details and waving at people in the VR environment and one participant (10%) expressed significant enjoyment of the VR scene with many positive and repeated vocalizations such as *“*it’s beautiful, waves coming in like soapy water,*”* “time to go paddling” and engagement with the VR environment, including wiggling their toes during the beach scene. This participant even made jokes about the scenes saying, “I’m going to drown if you don’t get me out of here” and “I think if I went here I would get lost”; the SDM confirmed that they were joking and the participant showed no signs of distress or agitation. During the post-VR interviews, 4 (40%) participants expressed enjoyment of VR therapy; 1 (10%) reported feeling bored but wanted to try VR again with different content and a lighter HMD, 1 (10%) had inconsistent feedback across sessions due to language impairments, 3 (30%) were unable to express any opinions because of language impairments or forgetting details of the experience, and 1 (10%) did not enjoy the experience because of side effects of nausea and dizziness.

The enjoyment caused by the VR session was often shared by caregivers or family members present during the VR session with the participants. In one of the 18 sessions (6%), the participant and 3 family members were all laughing so much that the nurse came to the room to see what was going on. During another session, the caregiver remarked that they had not seen anything (eg, television) hold the participant’s interest this well in over a year and recorded a video on their smartphone to share with friends and family. In another session, the participant was blowing kisses at the end of the session, which was enjoyed by the whole family who smiled and laughed along with the participant.

In almost two-thirds of the sessions (11/18, 61%), the RC perceived some to substantial participant relaxation from experiencing VR. Relaxation was perceived by observed behaviors during VR sessions and patient and caregiver feedback in post-VR interviews. Observed behaviors perceived as participant relaxation included deep, slow, and steady breathing, relaxed grip of the caregiver’s hand, and caregivers noting that the participant looks relaxed or calmer than usual*.* Relaxation was also confirmed verbally by participants, for example, in one of the 18 sessions (6%), the participant responded “yes, calming” when asked if they liked the session.

#### VR Content

Half of the participants (5/10, 50%) were able to provide verbal feedback about the VR content. When the 10 participants were asked what they liked most about the VR experience, 2 (20%) responded that they enjoyed water scenes the most, 1 (10%) responded that they enjoyed everything, 1 (10%) responded that they enjoyed the sounds the most, and 1 (10%) was not sure. When asked what other places they would like to see in VR, each of the 5 participants had a different answer. Despite these differences, all participants were asked about nature scenery. Table 4 provides detailed responses to questions related to the content of the VR experiences by participants.

**Table 4:** Virtual reality content participant feedback (N=10).

Question and participant number		Feedback
**What did you like most?**		
	P1	Unable to provide feedback
	P2	Unable to provide feedback
	P3	Unable to provide feedback
	P4	Unable to provide feedback
	P5	Provided different feedback during different sessions: “Sounds” Unable to provide feedback Unable to provide feedback “Very good” “Very soothing” Unable to provide feedback
	P6	“Not sure”
	P7	“Everything, liked it very much”
	P8	Provided different feedback during different sessions: “Swimming” “It was good” “The water”
	P9	“Beach”
	P10	N/A^a^—did not enjoy the session due to side effects
**Are there any other places you would like to see?**		
	P1	Unable to provide feedback
	P2	Unable to provide feedback
	P3	Unable to provide feedback
	P4	Unable to provide feedback
	P5	Provided different feedback during different sessions: “Park” Unable to provide feedback Unable to provide feedback Unable to provide feedback
	P6	“A combination of videos” “more nature–forest+birds”
	P7	“Liked water, seen too many forests, didn't like the forest”
	P8	Provided different feedback during different sessions: “The ocean” “Would like to see more animals like reindeer”
	P9	“Flowers, very nice, leaves” “Christmas trees+lights”
	P10	“Not sure”

^a^Not applicable.

## Discussion

### Principal Findings

The main finding of this pilot study was that the VR intervention was well accepted by the patient participants, consisting of individuals with a multitude of sensory, cognitive, and physical health conditions, including advanced dementia, limited mobility, and use of hearing and vision aids. In addition, despite many observed BPSD symptoms during their hospitalizations, none of the patients displayed aggressive or agitated symptoms during VR therapy, and none were actively averse to its application.

The Samsung Gear VR HMD was well tolerated by the participants and was reported to be comfortable by 7 of 10 participants (2 participants were unable to answer). A few challenges arose related to the HMD comfort and fit; the adjustable head straps were ill-fitting and resulted in the HMD slipping down the participant’s face.

VR exposure was considered acceptable by most (9/10, 90%) participants. For one participant who had nausea and dizziness, the RC noted that the participant was “actively looking around in all directions, head moving quite quickly left and right,” which may have contributed to these side effects. The outcomes indicate that in more than half of the immersive VR sessions, participants experienced enjoyment (10/18, 56%) and relaxation (11/18, 61%). Moreover, these effects were also felt by some caregivers and/or family members who were present. Most (7/10, 70%) participants expressed a desire to try immersive VR again, and there was interest in viewing more varied natural scenes and possibly personalized content. According to the participants’ suggestions, the VR film offering should be expanded to include a greater diversity of experiences such as live music scenes, scenes featuring people walking around, and scenes featuring animals.

Despite being conducted in sicker patients admitted to an acute care hospital, including moderate and advanced dementia, the outcomes of this study align with those of a previous feasibility study with 66 well, ambulatory older people with various physical and cognitive impairments, using a similar VR therapy exposure [22]. It is encouraging that VR therapy is feasible and has promising results across a spectrum of patients and environments, as clinicians from different hospital departments (eg, nephrology, intensive care, respirology) showed strong interest in evaluating VR intervention in their departments. Overall, patients with dementia appear to accept immersive VR, although there is a need to conduct rigorous studies and establish guidelines to ensure reliability and consistency in evaluating VR interventions.

### Limitations

The limitations of this research stem primarily from the small sample size and proof-of-concept approach characteristic of pilot studies. The design did not employ a control arm, and the sample size was not meant for statistical analysis of the significance of effects. With respect to outcomes regarding the feasibility of exposing patients with dementia to VR therapy in acute care hospitals, the main limitation was having no cases of patients in isolation because of infection control restrictions, patients with constant oxygen supplied through the nose, or patients fed through feeding tubes, as these are conditions that might be expected to influence the application of VR and would have been useful to study.

Another limitation of the study was the lack of validated instruments to measure the impact of nonpharmacological interventions in acute care hospitals. For the pilot study, we adapted existing scales (STAI, MiDAS), generally used in long-term care settings, to the relatively short-term stay in the acute care hospital.

In this study, we measured the impact on mood and symptoms (enjoyment and relaxation) based on the unblinded researchers’ observations. This may have introduced researcher bias.

### Conclusions

This was a small-scale pilot study carried out primarily to help identify feasibility issues in preparation for a large RCT designed to measure the impact of VR therapy on managing BPSD in acute care settings. The focus of the pilot was on testing various aspects of the proposed protocol (processes, methods, resources, etc) and validating the feasibility of using immersive VR technology for patients in all stages of dementia during their acute care hospital stay. The results demonstrate that VR therapy can be administered to patients living with dementia admitted to an acute care hospital, with patients accepting the hardware (equipment) and VR content very well with minimal, mild adverse effects. BPSD symptoms were not a barrier to using this equipment, and there were also no reports of audio frequency interference with hearing aids or with other medical devices while using VR.

These findings support conducting a large-scale RCT to investigate immersive VR therapy as a nonpharmacological intervention to manage BPSD in acute care hospitals. Particular interest should be given to people with more advanced stages of dementia (moderate to severe), as there are pervasive challenges in managing symptoms and improving the quality of life of these individuals using the current standard of care.

## Appendix

Multimedia Appendix 1 Recruitment flow diagram.

Multimedia Appendix 2 Informed consent form.

Multimedia Appendix 3 Demographic and baseline information.

Multimedia Appendix 4 Responses to pre- and postmood questions by session.

Multimedia Appendix 5 Participants’ ability to respond to mood questions.

## Abbreviations

BPSD: behavioral and psychological symptoms of dementia
EMR: electronic medical record
HMD: head-mounted display
IVR: immersive virtual reality
LoS: length of stay
MiDAS: Music in Dementia Assessment Scales
NPI: Neuropsychiatric Inventory
RC: research coordinator
RCT: randomized controlled trial
SDM: substitute decision maker
STAI: State-Trait Anxiety Inventory
VR: virtual reality
