# Supplementary figures and images for: Administering Virtual Reality Therapy to Manage Behavioral and Psychological Symptoms in Patients With Dementia Admitted to an Acute Care Hospital: Results of a Pilot Study
Source: JMIR Form Res. 2021 Feb 3;5(2):e22406. doi: 10.2196/22406 (PMC7889418; doi:10.2196/22406)

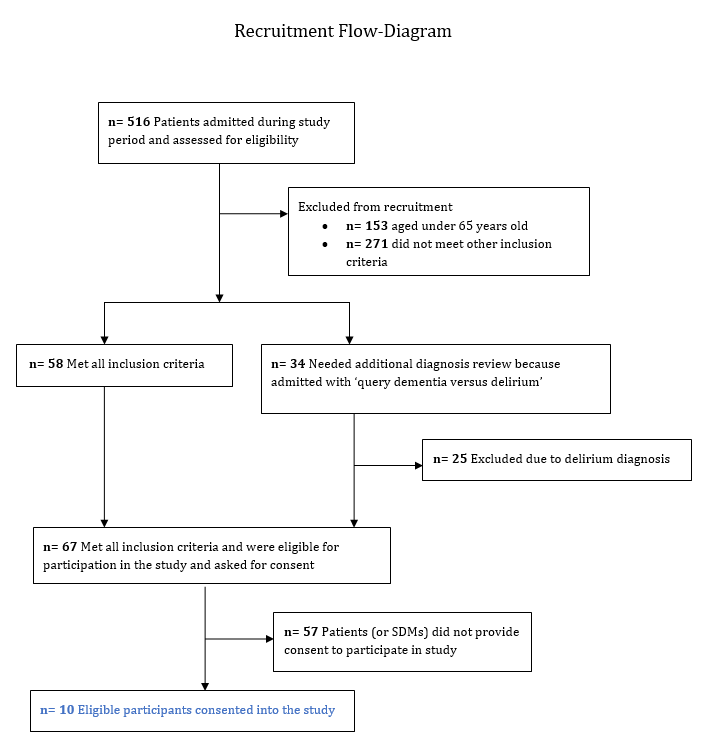

Supplement: Multimedia Appendix 1 [file formative_v5i2e22406_app1.png]
