# Supplementary material for: Administering Virtual Reality Therapy to Manage Behavioral and Psychological Symptoms in Patients With Dementia Admitted to an Acute Care Hospital: Results of a Pilot Study
Source: JMIR Form Res. 2021 Feb 3;5(2):e22406. doi: 10.2196/22406 (PMC7889418; doi:10.2196/22406)
Supplement: Multimedia Appendix 2 [file formative_v5i2e22406_app2.pdf]

MGH Hospital Letterhead

PATIENT INFORMATION AND INFORMED CONSENT FORM FOR RESEARCH PARTICIPATION

Study Title: VRx: Pilot Study to Design & Evaluate Virtual Reality based therapy for patients admited with dementia

Sponsor: Michael Garron Hospital

Investigators:

Primary Investigator: Dr. Christopher Smith (Christopher.Smith@tehn.ca)

Co-Investigators: Dr. Lora Appel (Lora.Appel@uhn.ca)

Dr. Jarred Rosenberg (Jarred.Rosenberg@tehn.ca)

INTRODUCTION

You have expressed an interest in participating in a research study regarding exposure to Virtual Reality therapy. This consent form provides you with information to help you make an informed choice. Please read this document carefully and ask any questions you may have. You may find it helpful to discuss the contents of this form with a friend or family member. All your questions should be answered to your satisfaction before you decide whether to participate in this research study. Please take your time in making your decision. The study staff will tell you about the study timelines for making your decision.

Your participation is entirely voluntary. Deciding not to take part or deciding to leave the study later will not result in any penalty or affect current or future health care. If you chose to withdraw from the study, information collected prior to your withdrawal may be used in our analysis, but no additional information will be collected or included in the study analysis and reporting.

Eligibility

To be eligible to participate in the study you must be:

- Over 65 years old
- Admitted to MGH
- Diagnosed with dementia

Background
Exposure to nature (sights and sounds) was shown to reduce depression, anxiety, aggression, and physiological distress. Virtual Reality (VR) presents a unique opportunity to transport patients away from their hospital environment that may be amplifying their distress, to calming natural environments (e.g. peaceful beach, green forest) that they would otherwise not be able to experience. VR head-mounted displays (HMDs) create the feeling of being truly present in the virtual world, seamlessly replacing the real world.

The purpose of this study is to explore novel ways to use Virtual Reality to facilitate relaxation and reduce feelings of distress associated with homesickness or social isolation during hospitalization. Elevated depression and anxiety during hospitalization can have a devastating impact on a patient's hospital stay: it may result in increases in medication, a lengthened course of treatment, impede recovery and participation in therapy, and create additional distress to the patient, their family members and care partners.
Our hypothesis is that VR experiences will facilitate relaxation and decrease feelings of distress during the hospital stay. Ultimately, we expect that for hospitalized people with dementia, who are missing their usual home environment, or receive minimal visitors and off-site stimulation, VR experiences will provide a better quality of life while in hospital undergoing rehabilitation therapy.
If successful, this may help develop a novel efficient VR based therapy.

What is the purpose of the study?
This study is an initial pilot / proof-of-concept to explore potential benefits of exposing patients with dementia admitted to an acute care hospital, to immersive visual and auditory displays enabled by VR technology.

METHODS AND PROCEDURES
You will take part in at least one activity (study) session and can opt to participate in additional sessions thereafter. The sessions will take place in a dedicated, safe hospital room. Hospital staff will take you to the study room at a time convenient to you and which does not interfere with your treatment activities. The initial session takes about 45 minutes. You will be sitting on a swivel chair that allows you to rotate around in a full circle, or an alternate chair of your choosing that is safe and approved by your healthcare providers. If you are unable to sit on a chair, you can watch the VR experiences from a hospital bed. Prior to starting the activity, a research team member will ask you some questions to determine how you feel

and your familiarity with VR technology. This will take about 15 minutes. The researcher will then help you put on a special VR headset, which is a device that resembles ski goggles and through which you can view VR experiences (films). You will be instructed to watch 3 to 5, short VR films of various lengths (3 or 5 minutes each) and different content (such as lake, forest). A research team member will observe your reactions during the session and will take notes (your movements, gestures, visual / audio feedback, comments you may make). At the end of the activity, the researcher will conduct an interview to find out how you feel after, and how you felt during the VR activity; any negative and/or positive feedback about the films, their content, their quality, the comfort and ease-of-use of the VR headset, and the experience in general. After the initial session, you may opt to participate in additional VR sessions over the course of your hospital stay. You will be asked to participate in brief interviews after each VR session.

There are no additional physical laboratory tests or procedures involved in this study; the intervention is only about wearing a special VR headset and watching Virtual Reality films and providing your feedback regarding this experience to the researcher. For the study we will also collect data from other medical tests conducted as part of your hospital standard of care. No impact on your routine care is expected during the study. The study sessions will accommodate your daily activities and will take place at a time mutually agreed mutually between treatments at the hospital.

CONFIDENTIALITY AND PRIVACY

Your data will be coded with a unique ID number. Identifying information about you will be stored confidentially in locked file cabinets and in computer files protected by a password. The only people who will know that you are a study subject are the members of the study staff. No identifying information about you will be disclosed by Michael Garron Hospital to others without your written permission, except: 1) if necessary to protect your rights or welfare (for example, if you are injured and need emergency care); or 2) if required by law. The study results may be published in a scientific journal or presented at scientific meetings, but your identity will remain de-identified. No personal references will be made at any time.

As part of continuing review of the research, your study records may be accessed on behalf of the Research Ethics Board. A person from the Michael Garron Hospital research ethics team may contact you (if your contact information is available) to ask

you questions about the research study and your consent to participate. The person accessing your file or contacting you must maintain your confidentiality to the extent permitted by law.

COMPENSATION

You will not have to pay for any of the procedures involved with this study.

There is no compensation for participating in this study.

POTENTIAL RISKS OF THE STUDY

Some studies warn that unfamiliar technology, such as Virtual Reality experiences, could be confusing to persons with cognitive impairment, and trigger disorientation or agitation. There is also possibility of feeling motion sickness, or nausea. However, from the few studies that have already been conducted on those with dementia, participants largely accepted the technology with very few negative consequences and indicated they would be open to participating with and engage in VR experiences in the future.

Trained clinicians will be available during the session to address any safety issues or concerns with the participants.

POTENTIAL BENEFITS FROM THE STUDY

There may be no direct benefit to you from taking part in this study. The immediate benefit during the intervention may be the enjoyment and relaxation produced by watching the immersive VR experiences. The benefit can also be awareness of this technology that can help you relax.
The information obtained from this study will contribute to testing whether it is safe, feasible for people with dementia to experience Virtual Reality, wearing a VR headset and watching immersive VR films. Further, it will help evaluate the willingness to use VR to experience simulated environments, such as exposure to nature, and whether this experience results in health benefits (such as reduced depression, stress, anxiety, wandering and falls, and increased feeling of presence and sense of self).

The results of this study may lead to the development of marketable treatments or devices. Any benefit from commercial products or services will remain with the study sponsor organization.

RIGHT TO ASK QUESTIONS AND TO WITHDRAW FROM THE STUDY

Your participation is voluntary, and you are free to withdraw from the study at any time. If you withdraw, we may ask you a few questions about your withdrawal, if you agree to discuss them with us. If you withdraw from the study, this will not affect any current or future Michael Garron Hospital treatment or care management.

Study information collected prior to your withdrawal will be coded with a unique ID number and may be used in our analysis, but no additional information will be collected or included in the study analysis and reporting.

If you have any questions concerning this study or if you experience a research-related problem or injury, contact:

Principal Investigator: Dr. Christopher Smith
Email: Christopher.Smith@tehn.ca
Telephone: 416-461-8272 x6252

If you have concerns or questions about your rights as a research participant in the study, feel free to call:

<Name of MGH REB Chair>, Chair of the Michael Garron Hospital Research Ethics Board at (416)nnn-nnnn ext. xxxx

MGH Hospital Letterhead

AGREEMENT TO PARTICIPATE ________

**Participant Consent to Allow Participation**

CONSENT: My signature below indicates-

- I have read the information provided above.
- The information has explained the study procedures, purpose, risks and benefits and I understand them.
- I understand that research carried out using my data by researchers at Baycrest, or their collaborators, may lead to the development of marketable treatments, devices, new drugs or patentable procedures. However, I understand that I will not be entitled to any benefits derived from any such commercial developments.
- I have been assured that confidentiality of my identifying information will be maintained.
- I have a right to withdraw myself from the study.
- I have been given the opportunity to ask questions and all my questions have been answered to my satisfaction.
- I have been given a signed copy of this form.
- By signing this form, I consent to participate in the research as described.

____

Name of Participant   Signature   Date Signed

**SIGNATURE OF PERSON OBTAINING CONSENT**

I have personally explained the research to the participant, and answered all of his/her questions. I believe that s/he understands the information described in this document and freely consents to participate.

____

Name of Person Obtaining Informed Consent (Print)   Signature   Date Signed
