# Supplementary material for: Administering Virtual Reality Therapy to Manage Behavioral and Psychological Symptoms in Patients With Dementia Admitted to an Acute Care Hospital: Results of a Pilot Study
Source: JMIR Form Res. 2021 Feb 3;5(2):e22406. doi: 10.2196/22406 (PMC7889418; doi:10.2196/22406)
Supplement: Multimedia Appendix 3 [file formative_v5i2e22406_app3.pdf]

Table 1. Demographic and Baseline information (N = 10).

Characteristic		Participants
Age in years, mean (SD)		86.5 (5.68)
**Gender**		
	Male, n (%)	2 (20)
	Female, n (%)	8 (80)
**Dementia Type**		
	Mixed dementia, n (%)	4 (40)
	Alzheimer's dementia, n (%)	4 (40)
	Vascular dementia, n (%)	1 (10)
	Frontotemporal dementia, n (%)	1 (10)
**Dementia Stages**		
	Mild, n (%)	2 (20)
	Moderate, n (%)	1 (10)
	Advanced, n (%)	4 (40)
	Unspecified, n (%)	3 (30)
**Delirium Diagnosis**		
	No delirium, n (%)	4 (40)
	Sub-acute, n (%)	1 (40)
	Acute, n (%)	3 (30)
	Chronic, n (%)	0 (0)
	Unspecified, n (%)	2 (20)
**Primary Language**		
	English, n (%)	5 (50)
	Greek/Macedonian, n (%)	3 (30)

	Bengali, n (%)	1 (10)
	Chinese, n (%)	1 (10)
**Current Living State**		
	Home alone, n (%)	3 (30)
	Home with family member(s), n (%)	1 (10)
	Retirement home/independent living, n (%)	1 (10)
	Assisted living/long term care, n (%)	4 (40)
	Other, n (%)	1 (10)
**Relationship Status**		
	Single, n (%)	3 (30)
	Married, n (%)	2 (20)
	Separated, n (%)	1 (10)
	Widowed, n (%)	3 (30)
	Other, n (%)	1 (10)
**Highest Level of Education**		
	Elementary school, n (%)	3 (30)
	High school or equivalent, n (%)	5 (50)
	College, n (%)	1 (10)
	Post-graduate degree, n (%)	1 (10)
**Vision devices**		
	Glasses, n (%)	8 (80)
	None, n (%)	2 (20)
**Hearing devices**		
	Hearing aid (both ears), n (%)	2 (20)
	None, n (%)	8 (80)

**Major auditory/visual condition**		
	Total deafness, n (%)	1 (10)
	Deafness (one ear), n (%)	1 (10)
	None, n (%)	8 (80)
**Head mobility**		
	Almost immobile, n (%)	0 (0)
	Limited, n (%)	1 (10)
	Normal, n (%)	9 (90)
**Body mobility**		
	Almost immobile, n (%)	1 (10)
	Limited, n (%)	8 (80)
	Normal, n (%)	1 (10)
**Mobility Aids**		
	Cane, n (%)	1 (10)
	Walker, n (%)	3 (30)
	Wheelchair, n (%)	3 (30)
	Multiple mobility aids, n (%)	3 (30)
