# Supplementary material for: Administering Virtual Reality Therapy to Manage Behavioral and Psychological Symptoms in Patients With Dementia Admitted to an Acute Care Hospital: Results of a Pilot Study
Source: JMIR Form Res. 2021 Feb 3;5(2):e22406. doi: 10.2196/22406 (PMC7889418; doi:10.2196/22406)
Supplement: Multimedia Appendix 4 [file formative_v5i2e22406_app4.pdf]

Table 5. Responses to pre- and post-VR therapy intervention mood questions by session (N = 18).

Session Date (dd-mmm-yyyy)	Participant Mood[a]															
	**Calm**		**Sad/Upset**		**Energetic**		**Lonely**		**Worried**		**Curious**		**Tired**		**Adventurous**	
	Pre-	Post-	Pre-	Post-	Pre-	Post-	Pre-	Post-	Pre-	Post-	Pre-	Post-	Pre-	Post-	Pre-	Post-
02-Aug-2018	Yes	Yes	No	No	Yes	Yes	No	No	No	No	Yes	Yes	No	No	No	Yes
17-Aug-2018	Mayb e	Yes	No	No	No	No	No	No	N/A	No	No	No	Mayb e	Little bit	No	N/A
21-Aug-2018	Yes	Yes	No	No	No	Little bit	N/A	No	N/A	No	Mayb e	Yes	Little bit	No	No	N/A
23-Aug-2018	N/A	N/A	N/A	N/A	Yes	Yes	N/A	N/A	N/A	N/A	N/A	N/A	No	No	N/A	N/A
24-Aug-2018	Yes	N/A	No	N/A	Little bit	Little bit	N/A	N/A	N/A	N/A	N/A	N/A	Little bit	Little bit	N/A	N/A
29-Aug-2018	Yes	Yes	No	No	No	No	No	No	No	No	No	Yes	No	No	No	No
05-Sep-2018	Yes	Yes	Yes	Yes	Yes	Yes	Yes	Yes	No	Yes	Yes	Yes	Yes	Yes	Yes	Yes
07-Sep-2018	Yes	Yes	Yes	N/A	Yes	Yes	Yes	Yes	Yes	Yes	Yes	Yes	Yes	Yes	Yes	Yes
10-Sep-2018	N/A	Yes	No	Yes	N/A	Yes	N/A	Yes	N/A	No	N/A	Yes	N/A	Yes	N/A	N/A
11-Sep-2018	Yes	Yes	Mayb e	No	No	No	May be	Yes	No	No	No	No	Yes	Yes	No	No
13-Sep-2018	N/A	N/A	N/A	N/A	No	N/A	N/A	N/A	N/A	N/A	N/A	N/A	Yes	N/A	N/A	N/A

14-Sep-2018	Yes	Yes	Yes	Yes	Yes	Yes	Yes	Yes	Yes	No	Yes	No	Yes	No	Yes	Yes
17-Sep-2018	No	Yes	Yes	No	No	Little bit	N/A	No	Yes	No	Yes	Yes	Yes	No	No	No
17-Sep-2018	Yes	Yes	Maybe	No	Maybe	Maybe	Yes	Maybe	No	No	No	No	No	No	Yes	Yes
19-Sep-2018	Yes	Yes	No	No	Little bit	Maybe	No	No	No	No	Maybe	Yes	Yes	No	Yes	Yes
20-Sep-2018	Yes	Yes	Yes	Maybe	Yes	N/A	Yes	Yes	Maybe	Maybe	Yes	Yes	Yes	Yes	Yes	Yes
20-Sep-2018	Maybe	Yes	No	No	Yes	Yes	No	No	Yes	Yes	Yes	Yes	Yes	Yes	No	No
24-Sep-2018	Yes	Yes	Maybe	No	No	No	No	Maybe	No	No	N/A	No	No	No	N/A	No

[a] Participant mood as identified by (in order of priority): participant, SDM/caregiver, or research coordinator.
