# Supplementary material for: Administering Virtual Reality Therapy to Manage Behavioral and Psychological Symptoms in Patients With Dementia Admitted to an Acute Care Hospital: Results of a Pilot Study
Source: JMIR Form Res. 2021 Feb 3;5(2):e22406. doi: 10.2196/22406 (PMC7889418; doi:10.2196/22406)
Supplement: Multimedia Appendix 5 [file formative_v5i2e22406_app5.pdf]

Table 6. Participant ability to respond independently to mood questions (N = 18).

Characteristic	**Participants**	
	Pre-intervention	Post-intervention
Calm, n (%)	11 (61)	11 (61)
Sad/Upset, n (%)	10 (56)	10 (56)
Energetic, n (%)	10 (56)	10 (56)
Lonely, n (%)	10 (56)	11 (61)
Worried, n (%)	10 (56)	11 (61)
Curious, n (%)	8 (44)	10 (56)
Tired, n (%)	10 (56)	11 (61)
Adventurous, n (%)	8 (44)	10 (56)
